# Poly (ε-caprolactone)-Based Scaffolds with Multizonal Architecture: Synthesis, Characterization, and In Vitro Tests

**DOI:** 10.3390/polym15224403

**Published:** 2023-11-14

**Authors:** Tainara de Paula de Lima Lima, Caio Augusto de Almeida Canelas, Joyce da Cruz Ferraz Dutra, Ana Paula Drummond Rodrigues, Rebecca Thereza Silva Santa Brígida, Viktor Oswaldo Cárdenas Concha, Fernando Augusto Miranda da Costa, Marcele Fonseca Passos

**Affiliations:** 1 Technological Development Group in Biopolymers and Biomaterials from the Amazon, Materials Science and Engineering Program, Federal University of Pará, Ananindeua 67130-660, PA, Brazil; taydilima@gmail.com; 2 Institute of Biological Sciences, Federal University of Pará, Belém 66075-110, PA, Brazil; caio.augusto.almeida1999@gmail.com (C.A.d.A.C.); fernando.amc@ufpa.br (F.A.M.d.C.); 3 Microbiology Department, Institute of Biological Sciences, Federal University of Minas Gerais, Belo Horizonte 31270-901, MG, Brazil; dutra.eng.enharia.ambiental@gmail.com; 4 Electron Microscopy Laboratory, Evandro Chagas Institute, Ministry of Health, Belém 66093-020, PA, Brazil; apdro.drigues@gmail.com (A.P.D.R.); rssb.1234@gmail.com (R.T.S.S.B.); 5 School of Chemical Engineering, Federal University of São Paulo, Diadema 09913-030, SP, Brazil; viktor.card.enas.c@gmail.com

**Keywords:** rotary jet spinning, alginate, hydrogel, pracaxi oil, *Pentaclethra macroloba*, tissue engineering

## Abstract

Tissue engineering is vital in treating injuries and restoring damaged tissues, aiming to accelerate regeneration and optimize the complex healing process. In this study, multizonal scaffolds, designed to mimic tissues with bilayer architecture, were prepared using the rotary jet spinning technique (RJS scaffolds). Polycaprolactone and different concentrations of alginate hydrogel (2, 4, and 6% *m*/*v*) were used. The materials were swollen in pracaxi vegetable oil (PO) (*Pentaclethra macroloba*) and evaluated in terms of surface morphology, wettability, functional groups, thermal behavior, crystallinity, and cytotoxicity. X-ray diffraction (XRD) showed the disappearance of the diffraction peak 2θ = 31.5° for samples from the polycaprolactone/pracaxi/alginate (PCLOA) group, suggesting a reduction of crystallinity according to the presence of PO and semi-crystalline structure. Wettability gradients (0 to 80.91°) were observed according to the deposition layer and hydrogel content. Pore diameters varied between 9.27 μm and 37.57 μm. Molecular interactions with the constituents of the formulation were observed via infrared spectra with Fourier transform (FTIR), and their influence was detected in the reduction of the maximum degradation temperature within the groups of scaffolds (polycaprolactone/alginate (PCLA) and PCLOA) about the control. In vitro tests indicated reduced cell viability in the presence of alginate hydrogel and PO, respectively.

## 1. Introduction

Tissue engineering (TE) is a branch of multidisciplinary research that has emerged as an exciting alternative to overcome problems involving the scarcity of resources to transplant damaged organs and tissues [1]. In this technique, the patient’s cells are cultivated and placed on a three-dimensional (3D) support (scaffolds), contributing to the formation of new tissue and reducing the rejection rates of the biological system [2,3]. Scaffolds are three-dimensional structures designed to mimic the natural extracellular matrix and provide physical and mechanical support for cell growth. These materials can be developed into multizonal architectures made of biocompatible polymers and hydrogels and loaded with bioactive compounds essential for regeneration [4]. A multizonal material presents more than one layer in its composition to imitate the heterogeneity of the tissue’s native structure. It can be constituted by joining two or more materials, presenting an innovative approach as an interface between different tissues, composite organs, and musculoskeletal junctions. In tissue engineering, multizonal scaffolds are studied in vascular and bone-cartilaginous tissues, among others. For example, Kang, Zeng, and Varghese (2018) developed multizonal scaffolds using hydrogels and biomineralized materials for bone tissue engineering [5]. A wide range of polymeric biomaterials can be used as scaffolds, especially poly (ε-caprolactone) (PCL) [6]. This polymer, from the family of aliphatic polyesters, is widely used in the medical field as it offers certain advantages, such as biocompatibility, great miscibility, compatibility with other types of polymers, solubility in various organic solvents, and ease of manufacturing to obtain different shapes (films, fibers, and pellets) and mechanical properties to be applied in specific areas [7]. One of the processing methods is the rotary jet spinning technique. This low-cost method uses centrifugal force to obtain PCL fibers, mimicking the extracellular matrix and favoring cell adhesion and proliferation for potential use in tissue engineering [8,9]. However, PCL has low hydrophilicity, and this disadvantage can be altered by combining with hydrophilic materials, such as hydrogels, thus generating new or improved materials by the synergism of physicochemical and biological properties.

Hydrogels are crosslinked hydrophilic polymers with a three-dimensional structure that demonstrate similarities with the physiological properties of living tissues, e.g., swelling when exposed to water or fluids of biological origin, which favors cellular inoculation [10]. Furthermore, they are intended for use in cell cultures and can be made from a wide range of natural and synthetic materials, providing a broad spectrum of chemical properties [11]. One of the hydrogels widely used in tissue engineering is alginate. Alginate is a natural polymer from brown algae and is also produced by some bacteria, such as *Pseudomonas aeruginosa* [12]. Its composition contains repeated units of ß-D-mannuronic acid and α-L-guluronic acid, which can influence the properties and physicochemical characteristics of the alginate, producing more rigid or flexible materials [13]. Similar to PCL, alginate can be used in different formats (film, membrane, sponges, nanofibers, etc.). However, for tissue engineering applications, its hydrogel structure is often desired. Alginate hydrogels present satisfactory characteristics, such as hydrophilicity, elasticity, and softness; fluid absorption capacity (exudate); non-toxicity; non-irritability to the skin, and do not cause secondary injuries when removed [14]. The sodium alginate polymer can instantly form hydrogels via ionic crosslinking in the presence of bi- or trivalent ions due to the strong interaction between alginate crosslinking ions [15]. This crosslinking leads to improved mechanical strength, vapor barrier properties, etc. Moreover, this hydrogel can be incorporated with other polymers, such as PCL, increasing its physical/chemical, mechanical, and biological efficiency [16]. Such characteristics can also be associated with bioactive ingredients, preventing local infections and favoring more significant cell proliferation and adhesion in materials composed of PCL alginate. An example is using vegetable oils from the Amazonian biodiversity, such as pracaxi oil from the *Pentaclethra macroloba* species. The presence of triterpene saponins, sterols, tannins, oleanolic acid, and fatty acids demonstrated the potential of this oil in the healing of wounds and burns [17] and the potential use for incorporating the oil into drug delivery systems [18]. The chemical composition of the aqueous extract of pracaxi oil, with high levels of behenic acid and oleic acid, has also been reported in treating diseases [19]. Its fatty acids are essential natural bioactives with anti-inflammatory properties and can act against bacterial infections. However, it should be noted that there are few data in the literature on the in vitro assay of pracaxi oil, and no studies have been carried out using multizonal structures loaded with this natural oil from the Amazon, making this a pioneering work for tissue engineering. Therefore, this work aimed to obtain and physicochemically characterize multizonal scaffolds based on rotary jet-spun PCL, alginate hydrogels, and pracaxi oil. An in vitro cell viability study was also conducted to evaluate the formulation components’ influence on the materials’ cytotoxicity.

## 2. Materials and Methods

### 2.1. Materials

Poly (ε-caprolactone) (PCL) (MM = 80,000 g/mol), calcium chloride (MM = 110.98 g/mol and purity content ≥93%), sodium alginate (ALG; CAS number9005-38-3), solution phosphate saline buffer (PBS) (10-fold concentrate), cell growth assay kit (MTT-3-(4, 5-dimethylthiazolyl-2)-2, 5-diphenyltetrazolium bromide), and dimethylsulfoxide (DMSO) were supplied by Sigma-Aldrich (Cotia, SP, Brazil). Dulbecco’s modified Eagle medium (DMEM) and fetal bovine serum (FBS) were obtained from Gibco. Pracaxi oil (PO) was purchased by Amazon Oil (Belém, PA, Brazil), and its physicochemical characteristics can be seen in Table 1. Dichloromethane P.A (MM = 84.93 g/mol and purity content ≥99.5%) and hydrochloric acid P.A (MM = 36.49 and purity between 36.50% and 38.00%) were obtained from Neon Comercial (Suzano, SP, Brazil). Sodium hydroxide P.A (MM = 40.00 g/mol and purity content = 97%) was provided by Êxodo Ciência (Sumaré, SP, Brazil).

### 2.2. Preparation of Solutions

The aliphatic polyester polymeric solution, with a concentration of 20% *m*/*v*, was prepared by dissolving the PCL pellets in dichloromethane. Polysaccharide (ALG) solution was obtained by solubilizing the powder in distilled water at 70° C at 3 different concentrations: 2, 4, and 6% *w*/*v*. As a plasticizer at 40% *w*/*w*, glycerin was added to the ALG solutions according to methodology adapted from Yang et al. (2016) [20]. Calcium chloride solution was prepared at room temperature at 4% *w*/*v* in distilled water. All solutions were kept under magnetic stirring until complete solubilization.

### 2.3. Formation of Scaffolds via Rotary Jet Spinning System

**Figure 1:**
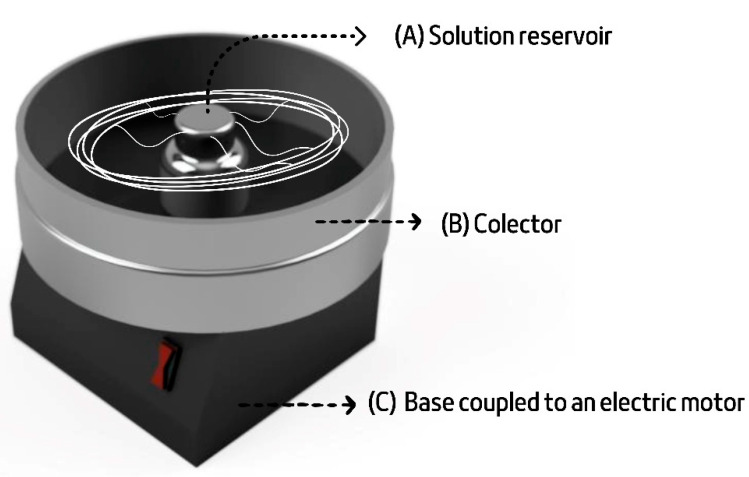
Schematic representation of the rotary jet spinning system.

PCL-based scaffolds (sample PCL—0) were produced by the rotary jet spinning technique (RJS scaffolds), according to the schematic diagram in Figure 1. The equipment developed in this study consisted of a central reservoir with four outlet capillaries (Ø = 0.5 mm) used to expel the aliphatic polyester solution (20 % *w*/*v*; PCL—0) (a), a collector with a cylindrical shape for depositing the fibers formed by extrusion (b), and a circular base coupled to a rotor with a rotating speed of 19,200 rpm (c). The distance between the collector and the central reservoir and the volume of PCL solution added to the reservoir were kept constant at 50 cm and 4 mL, respectively. The process was carried out in batches, forming four layers of material at room temperature. The scaffolds were dried in an oven (SolidSteel (SSD 40 L)) to eliminate possible residual solvents for 48 h at 40 °C and then were cut into a size of 1 × 1 cm. Next, PCL-ALG samples (PCLA group) were obtained by ionic gelation of the ALG (2, 4, and 6% *w*/*v*) on one of the superficial sides (SA side) of the rotary jet-spun PCL—0, using steps of controlled immersion of the PCL—0 in the solutions of ALG (5 min) and calcium (60 min), respectively. The ionic gelation process consisted of the electrostatic interaction between the physical crosslinker and the alginate, which possessed opposite charges. Hydroxyl and carboxyl groups of ALG non-covalently interacted with divalent cations of calcium chloride to form the hydrogel on the surface of PCL—0 scaffolds.

To prepare the PCLOA group, the PCLA samples were immersed in 5 mL of pracaxi oil for 24 h. Excess oil was removed on absorbent paper, and the materials were stored under refrigeration (4 °C) for further characterization. The composition and identification of the samples are described in Table 2.

### 2.4. Physical/Chemical Characterization

The fatty acid profile of pracaxi oil was determined by gas chromatography (GC), and the analyses were performed under conditions used in previous studies [21]. Thus, the methyl esterification of fatty acid was realized using the boron trifluoride (BF_3_) method described in the International Standardization Organization [22]. The time of peak retention was compared to the standards of respective fatty acids to obtain qualitative composition, and the AOCs Ce 2-66 official method was used to obtain the quantitative composition by peak area normalization [23]. The morphology of the materials was assessed in a scanning electron microscope (SEM) (Jeol JSM-6610LV), operating at an accelerating voltage of 1 kV, with magnifications of 500× and 1000×. Before SEM imaging, RJS scaffolds were covered with a thin layer of gold (Denton Vacuum, Desk V model). Inter/intramolecular interactions were investigated using a Fourier transform infrared spectrophotometer (FTIR) (Shimadzu, model IRPrestige-21), in the range of 4000 to 650 cm^−1^ and a resolution of 4 cm^−1^. Thermal stability was evaluated using a thermogravimetric analyzer (Shimadzu, DTG-60H) in the range of 25 to 650 °C, inert nitrogen atmosphere at 50 mL/min, and heating rate at 10 °C/min. An X-ray diffractometer (Bruker, D8 Advance DAVINCI) equipped with Cu Kα radiation was used to evaluate the crystallographic structures of the scaffolds. The analyses were evaluated at 30 kV and 30 mA, 2θ interval (5–40°), and at a 2°/min speed.

The surface wettability of the scaffolds was investigated by apparent contact angle (θ) measurements using the sessile drop mode at room temperature. The images were captured by a Nikon b500 16 mp/40× camera, and the drop contour and contact angle were analyzed using the Image J program [21]. Values were expressed graphically as mean and standard deviation (n = 3). For this purpose, the contact angle data were analyzed using the GraphPad Prism 7^®^ software.

### 2.5. In Vitro Assay

#### 2.5.1. Cultivation of 3T3 Fibroblasts

The cytotoxicity assay used the murine fibroblasts of the BALB/c 3T3 lineage, clone A31, from the Rio de Janeiro Cell Bank (BCRJ:0047). The cells were cultivated in DMEM medium supplemented with 10% fetal bovine serum (FBS) and kept in an atmosphere with 5% CO_2_ at 37 °C until the culture reached 80% confluency. At that moment, the fibroblasts were washed with sterile phosphate saline solution (PBS) and subjected to the action of 0.05% trypsin, being incubated at 37 °C for 3 min, for the detachment of these cells from the surface of the bottle. Then, DMEM medium supplemented with SBF was added to neutralize the trypsin action. The bottle contents were then transferred to a tube and centrifuged (1500 RPM/5 min/4 °C). The supernatant was discarded, and the cells were resuspended in PBS for washing, being centrifuged again under the same conditions mentioned above. The supernatant was again discarded, and the pelleted cells were resuspended in DMEM, then counted, and their concentration adjusted to 1 × 105 cells/well.

#### 2.5.2. Cytotoxicity of RJS Scaffolds in 3T3 Fibroblasts by the Thiazolyl Blue (MTT) Method

Cellular cytotoxicity was evaluated using the MTT assay, which determines viability by mitochondrial activity. Mus musculus embryo BALB/3T3 clone A31 cells were added onto the RJS scaffolds in a 24-well plate and incubated for about one hour at 37 °C under 5% CO_2_. Then, DMEM was added, supplemented with 10% SBF in each well, and the set (cells and scaffolds) was again incubated under the same conditions mentioned above for 24 h. After this period, the supernatant was removed, and 0.5 mg/mL of MTT diluted in PBS was added to the wells. The plate was incubated for 3 h in a 5% CO2 atmosphere at 37 °C. After incubation, the MTT solution was removed, the wells were washed with PBS, and the supernatant was removed. Subsequently, 200 μL of dimethylsulfoxide (DMSO) was added to each well, and the plate was kept in agitation for 10 min. The final solution was transferred to a 96-well plate and read by an EZ Read 2000 Microplate Reader/Biochrom spectrophotometer with a wavelength of 570 nm. The generated absorbance values were used to determine the percentage of cell viability, considering the control as 100% viable cells. The experiment was carried out in triplicate, using dead cells exposed to 10% paraformol as reaction control, in addition to the control group with live cells.

### 2.6. Statistical Analysis

The Shapiro–Wilk test was used to assess the normality of the sample population to assay contact angle. A value of *p* < 0.05 indicated rejection of the null hypothesis (the sample population followed a normal distribution). Statistical differences between groups were evaluated using the ANOVA statistical test and Tukey’s test. For in vitro tests, all data points were assessed by GraphPad Prism 7^®^ using a *t*-test with a 95% confidence level. The results were considered statistically significant to *p* < 0.05 (*).

## 3. Results and Discussion

### 3.1. Fatty Acid Composition of Pracaxi Seed Oil (Pentaclethra macroloba)

The chemical composition of PO fatty acids can be seen in Table 3. A total of twelve fatty acids were identified, with oleic acid (53.68%), behenic acid (13.74%), and linoleic acid (12.61%) being the major components, with considerable importance in several biological processes, e.g., protection from wound injury skin accelerating the healing process and tissue regeneration [24]. Oleic acid is known to have anti-inflammatory and anti-oxidant properties [25]. Studies also suggest its importance in reducing local inflammation and cell communication, which may be crucial in coordinating tissue regeneration processes [26]. Behenic acid may play a role in maintaining the integrity of cell membranes and in the formation of protective barriers [18]. Linoleic acid acts in the production of prostaglandins and other bioactive substances, which are involved in the inflammatory response and the healing process [27]. Simons, Banov, and Banov (2015) demonstrated the importance of PO fatty acids in closing skin lesions, optimizing the healing process, and helping wound re-epithelialization [17].

The presence of lauric acid (0.77%), myristic acid (0.72%), palmitic acid (2.42%), margaric acid (1.44%), stearic acid (3.145%), nonadecanoic acid (0.14%), arachidonic acid (1.34%), tricosanoic acid (0.12%), and lignoceric acid (9.82%) were also identified, which can contribute to the maintenance, protection, and restoration of skin tissue [28,29]. Similar results were also found by Teixeira et al. (2020) [30] and Morguette et al. (2019) [31].

### 3.2. Production of RJS Scaffolds and Morphological Characteristics by SEM

**Figure 2:**
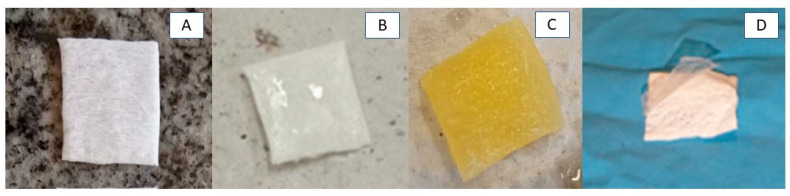
Macroscopic images of rotary jet-spun scaffolds, respectively: (**A**) PCL—0; (**B**) PCLA group (PCLA—2% and PCLA—4%); (**C**) PCLOA group; (**D**) PCLA—6%.

**Figure 3:**
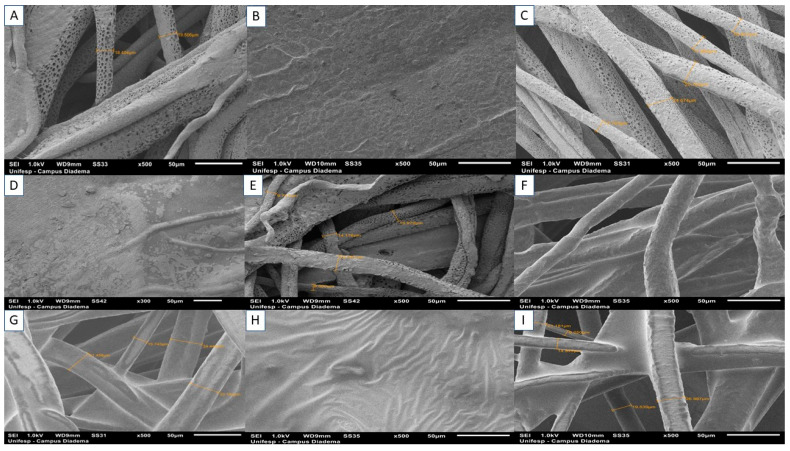
Micrographs (SEM) of the rotary jet-spun scaffolds at 500× magnification, respectively: (**A**) PCL—0 SEM; (**B**) PCLA—2% SEM (SA); (**C**) PCLA—2% SEM (SB); (**D**) PCLA—4% SEM (SA); (**E**) PCLA—4% SEM (SB); (**F**) PCLOA—2% SEM (SA); (**G**) PCLOA—2% SEM (SB); (**H**) PCLOA—4% SEM (SA); (**I**) PCLOA—4% SEM (SB).

Several factors affect the effectiveness of polymers for use as 3D matrices, including the methods of manufacture and their chemical compositions [32]. For example, the rotary jet spinning technique can form micro/nanofibers and dense or porous scaffolds. The type of solvent, rotation speed, and spinning distance can modulate such characteristics [33]. Figure 2A–D show the macroscopic images of the RJS scaffolds, and the micrographs can be seen in Figure 3A–I. An accentuated shine is observed on the surface of the samples from the PCLA group (Figure 2B), attributed to the presence of ALG. The PCLOA group, on the other hand, showed a yellowish color, typical of PO (Figure 2C). Figure 2D shows the low interfacial adhesion between ALG and PCL, leading to detachment of the hydrogel in all samples with 6% *m*/*v* of the polysaccharide (PCLA—6% and PCLOA—6%). According to Yu et al. (2023) [4], this behavior is one of the main limitations of multizonal bilayer materials. It occurs due to the weak bond strength between the surfaces, which leads to their detachment. Thus, the PCLA—6% and PCLOA—6% samples were discarded for the other characterizations.

Figure 3A (PCL—0) shows the formation of a porous and microfibrous structure, with diameters ranging from 18.40 to 19.50 μm. Several studies have demonstrated that the choice of solvent directly influences the structure of materials obtained by the rotary jet spinning technique [34]. Dichloromethane contributes to this morphology as it has a low boiling point and high volatility. Modolo et al. (2023) state that porous and fibrillar structures facilitate vascularization, nutrient diffusion, adhesion, and cell growth [35]. Similar results can be found in the work of Altun et al. (2022) [36]. For the scaffolds of the PCLA and PCLOA groups, it was possible to perceive the influence of alginate on the surface in contact with the ionic gelation process (SA). The pores were closed, and the hydrogel covered the fibers, resulting in dense and rough morphologies. On the B side (SB), on the other hand, fiber maintenance was observed in samples PCLA—2% (Figure 3C), PCLA—4% (Figure 3E), PCLOA—2% (Figure 3G), and PCLOA—4 % (Figure 3I), with diameters ranging from 15.74 to 34.44 μm; 33.27 to 37.57 μm, 13.79 to 25.70 μm, and 9.27 to 23.08 μm, respectively. For the PCLA group, porosity was also evident, suggesting that the ALG was not homogeneously absorbed throughout the entire depth (four layers) of the RJS scaffolds. However, closure of the pores within the fibers was observed for the PCLOA groups, which may be associated with the addition of PO to the scaffolds. Similar results were obtained by [21].

### 3.3. Fourier Transform Infrared Spectroscopy (FTIR)

**Figure 4:**
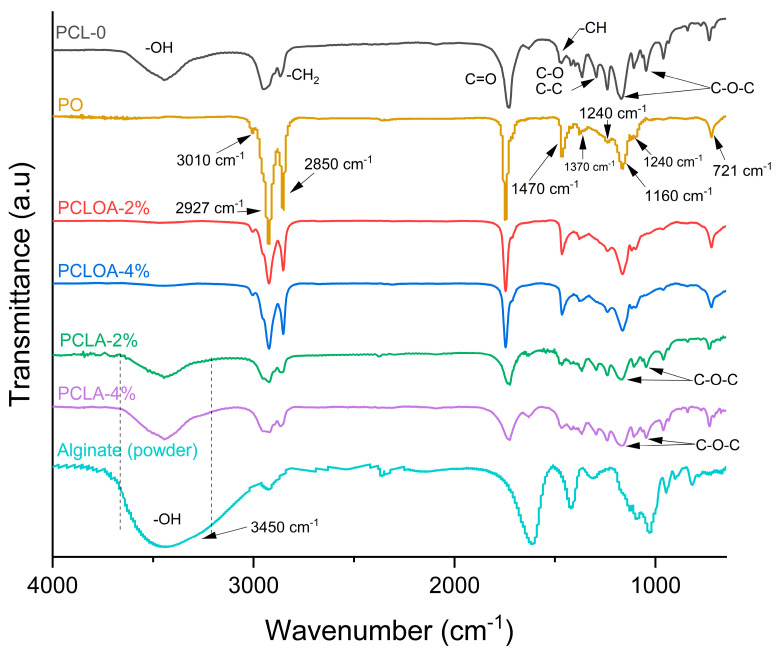
FTIR spectra of PCL—0, PCLA—2%, PCLA—4%, PCLOA—2%, PCLOA—4%, and powdered sodium alginate.

Figure 4 shows the FTIR spectra of the PO, the sodium alginate powder, and the RJS scaffolds (PCL—0, PCLA—2%, PCLA—4%, PCLOA—2%, and PCLOA—4%). For PO, a low-intensity band at 3010 cm^−1^ was verified, referring to the elongation of non-conjugated C-H double bonds, symmetrically disubstituted in the cis position. High-intensity bands were identified at 2927 and 2850 cm^−1^, corresponding to the HC= and –CH_3_ groups, respectively. At 1747 cm^−1^, carbonyl elongation and vibration of the C-O bond can be seen at 1240, 1160, and 1100 cm^−1^. At 1470 and 1370 cm^−1^, bands of moderate intensity were identified related to the axial deformation of aliphatic C-H groups and a 721 cm^−1^ out-of-plane CH deformation [18].

A PCL—0 sample showed the following typical polycaprolactone bands: a band in the range of 3637 to 3326 cm^−1^, which can be attributed to the vibration of stretching and deformation of the hydroxyl groups (O-H) as a function of water absorption and coordination with the water [37]; at 2942 cm^−1^, which can be referred to as the asymmetric stretching of the -CH_2_ group; at 2868 cm^−1^, related to the symmetric stretching of the -CH_2_ group; at 1726 cm^−1^, due to the presence of the carbonyl ester group (C=O), with consequent stretching vibration of -CO [38]; at 1474 cm^−1^, due to C-H vibration; at 1298 cm^−1^, due to C-C and C-O stretching; at 1169 cm^−1^, attributed to the asymmetric stretching of the C-O-C bond; and at 1040 cm^−1^, due to C-O-C stretching vibration [39]. For sodium alginate (powder), bands were identified at 3378 cm^−1^, representing the stretching vibration of the hydroxyl group O-H; at 2929 cm^−1^, attributed to the symmetric stretching mode for CH_2_ [40]; at 1610 cm^−1^, referring to the COO- asymmetric stretch; at 1413 cm^−1^, attributed to the C-OH group; at 1121 cm^−1^, typical of the vibrations of the C-O/C-C groups; at 1087 cm^−1^, related to the C-O-C cluster; and at 1026 cm^−1^ and 944 cm^−1^, referring to the stretching of the C-O group, related to the residual mannuronic and guluronic acids in the composition of the alginate [41,42].

Spectra of PCLA—2% and PCLA—4% scaffolds showed similar profiles, with the presence of the band attributed to the hydroxyl group (present in alginate and PCL) in the range of 3650 to 3200 cm^−1^, in addition to changes in the bands of stretching vibrations C-O-C and C-C. The sodium alginate crosslinking process on the surface of PCL—0 scaffolds can explain the latter. Divalent Ca^2+^ ions (from calcium chloride) react with hydrophilic groups –COO and -OH in the scaffold structure, forming the calcium alginate hydrogel/three-dimensional polymeric networks [43]. However, increasing the alginate content (PCLA—2% to PCLA—4%) increases the intensity of the OH band due to the carboxyl group. Nevertheless, the hydroxyl band is reduced in the PCLOA—2% and PCLOA—4% scaffolds, and the band’s appearance at 3010 cm^−1^ is typical of PO. The addition of pracaxi oil can lead to the formation of hydrogen bonds between the hydrogen atom of the hydroxyl group of the polysaccharide carboxyl group and the carbonyl oxygen of pracaxi oil, reducing the intensity of the OH groups identified by FTIR, which suggests molecular interactions between the components of the scaffold formulation.

### 3.4. Contact Angle Assay

**Figure 5:**
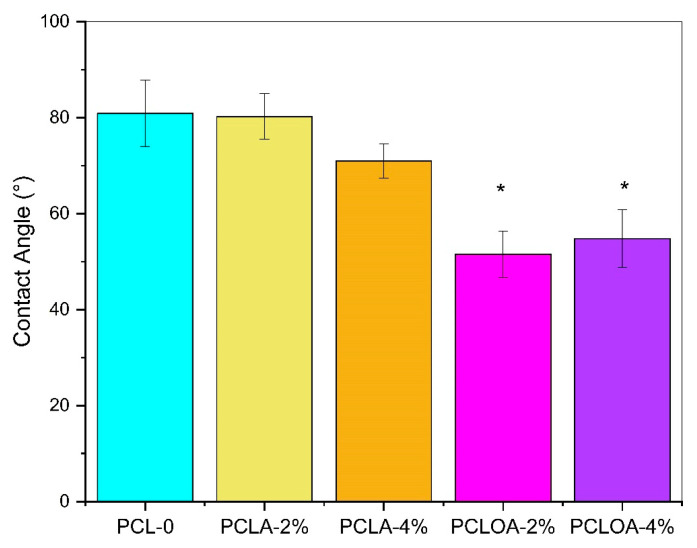
The contact angle (SB) of PCL—0, PCLA—2%, PCLA—4%, PCLOA—2%, PCLOA—4%, RJS scaffolds. Results expressed as mean ± standard error of the mean. The symbol * represents *p* < 0.05 in relation to the control group.

Contact angle at 0° was observed on the A (SA) side of the RJS scaffolds, for the PCLA and PCLOA group, indicating complete wettability of the materials and superhydrophilic character. The surface adhesion of the polysaccharide can explain this behavior. Similar results were also found by Echeverria Molina et al. (2023) [44]. For side B (SB), the following contact angles were found: 80.91° ± 6.99° for PCL-0; 80.26° ± 4.78° for PCLA—2%; 71.01° ± 3.60° for PCLA—4%; 51.57° ± 4.84° for PCLOA—2%; and 54.80° ± 5.99° for PCLOA—4% (Figure 5). Statistically, no significant difference (*p* > 0.05) in wettability was observed within groups and between the control group (PCL—0) and the ALG-containing scaffolds (PCLA—2% and PCLA—4%). 

However, different wettability profiles of RJS scaffolds between the faces of the materials were observed. The alginate was not wholly absorbed on the B side, and the hydrophilic characteristic (θ < 90°) was predominant in the processing of polycaprolactone. Research [45] has demonstrated that fibrous structures, based on polyester, have contact angle values above 100° and hydrophobic character [46]. Nevertheless, for polymers with low hydrophilicity, the presence of pores in the morphology optimizes liquid absorption [47]. Thus, the rotary jet spinning process favored the wettability of RJS scaffolds due to the formation of inter/intramolecular pores [48], as demonstrated in the SEM micrographs. Similar results were also found by Wang et al. (2021) [47].

On the other hand, between the samples from the PCLOA group and the PCLA group and between the PCLOA group and the PCL—0 scaffold, a statistically significant difference in wettability was observed. The scaffolds showed a more hydrophilic surface characteristic. The presence of PO significantly altered the surface wettability (*p* < 0.05). The optimization of hydrophilicity due to the addition of vegetable oils was also observed by Paranhos et al. (2022) [49]. The RJS scaffolds with the hydrogel and PO layer showed different wettability profiles during their depth (four layers). The closer to the A side (ALG), the more hydrophilic the surface, and this behavior can characterize the samples as multizonal scaffolds discontinuous, with varied functionalities on the surface and in the transition zone between the polymers [4]. For applications in soft tissue regeneration, for example, hydrophobic or less hydrophilic regions can act as a protective barrier against possible infections of microorganisms to the injured site. Moreover, the areas with a hydrophilic character (side A) can keep the environment humid, with good exudate absorption capacity [50,51,52]. In osteochondral repairs, scaffolds with multizonal bilayer architecture show an interesting strategy to mimic tissue architecture: the hydrogel acts as cartilage, and the PCL acts as a substitute for the bone layer [4].

### 3.5. X-ray Diffraction (XRD)

**Figure 6:**
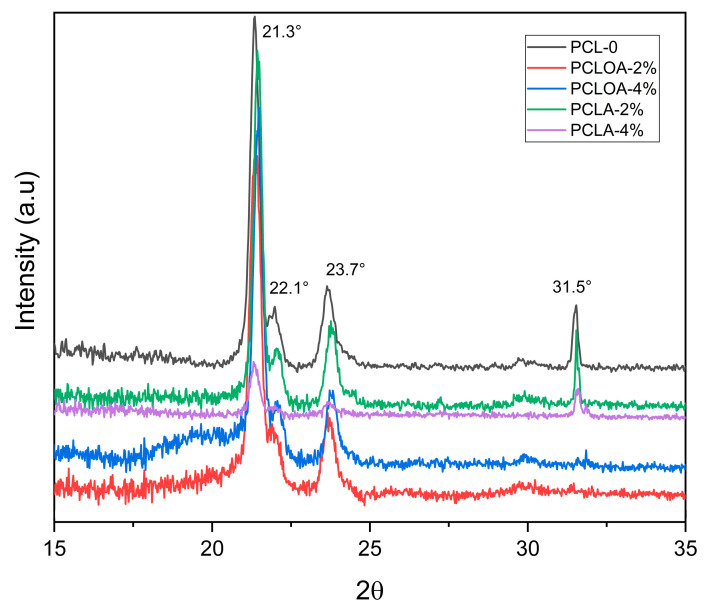
X-ray diffractogram of the RJS scaffolds of PCL—0, PCLOA—2%, PCLOA—4%, PCLA—2%, and PCLA—4%.

Figure 6 shows the X-ray diffractogram of the RJS scaffolds. In all samples, characteristic PCL peaks at 2θ = 21.4°, 22.1°; 23.7°, and 31.6° were observed and corresponded, respectively, to the diffraction planes (110), (111), and (200) of the orthorhombic crystalline structure of polyester [38]. This result is in line with the work by Rodenas-Rochina et al. (2015) [53]. In PCLOA—2% and PCLOA—4% samples, the diffraction peak 2θ = 31.5° disappeared. PO interferes with the reduction of crystallinity and can influence molecular interactions (strength and nature of bonds) in cell material, influencing how proteins and other molecules involved in cell adhesion interact with the surface.

### 3.6. Thermogravimetric Analysis (TGA)

**Figure 7:**
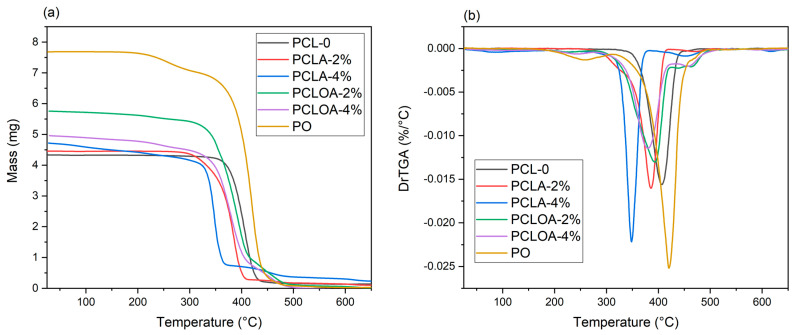
TGA (**a**) and DrTGA (**b**) from PCL—0, PCLA—2%, PCLA—4%, PCLOA—2%, and PCLOA—4% samples.

Figure 7a,b show the mass loss and its derivative for the studied scaffolds, respectively. The maximum decomposition (Tdmax), onset degradation temperatures (Tonset), mass loss, and the percentage of residues are given in Table 4. A mass loss of 95.16% at the maximum temperature of 406.9 °C (Tonset = 324.7 °C) was observed in the PLC—0 sample, justified by the breaking of the polycaprolactone main chain by the pyrolysis reaction [21]. In the case of pracaxi oil, there were two levels of mass loss, 8.75% and 89.56%, at temperatures of 258.07 °C and 420.31 °C (Tdmax), respectively, with a total mass loss of 98.31% (Table 4). The initial mass loss corresponded to the elimination of water and volatile compounds. The second level of degradation referred to the breakdown of the long chain of fatty acids in the oil [18]. Similar results can be found in the work of Costa et al. (2014) [54]. In the PCLA—2% sample, two levels of mass loss were observed, 94.16% and 2.25%, at the maximum temperatures of 385.77 °C and 472.28 °C, respectively. For PCLA—4%, however, three levels of mass loss (13.78%, 70.12%, and 7.84%) were observed at maximum temperatures of 85.9 °C, 348.13 °C, and 451.59 °C. As the alginate content increased, the first stage became pronounced, probably indicating a more significant loss of water (moisture) and volatiles due to the hydrophilic nature of the polysaccharide. The other events were associated with the rupture of the main polymer chains and the possible formation of carbonaceous residue [55]. Three mass loss events were observed in the PCLOA group scaffolds (PCLOA—2% and PCLOA—4%). The Tdmax were 393.46 and 379.06 °C, and their respective mass losses were 97.40% and 98.21%. The addition of oil and alginate hydrogel, therefore, led to a decline in the thermal stability of the RJS scaffolds.

### 3.7. Cytotoxicity of RJS Scaffolds in 3T3 Fibroblast

**Figure 8:**
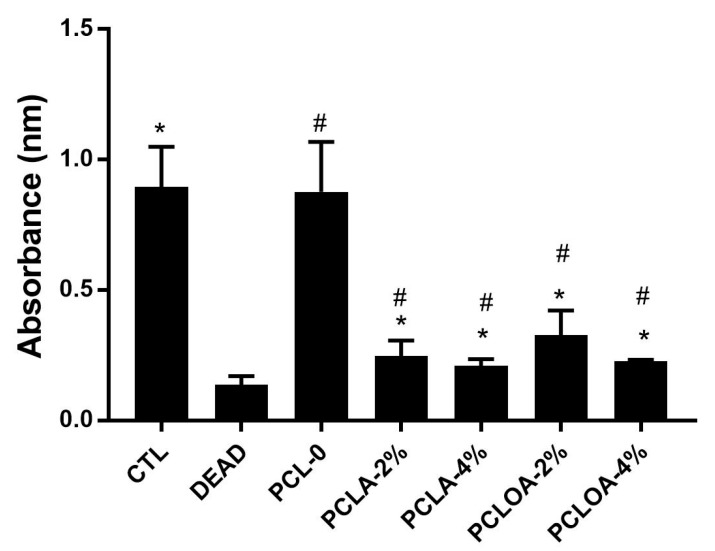
Cell viability of 3T3 fibroblasts treated with PCL—0, PCLA (PCLA—2% and PCLA—4%), or PCLOA (PCLOA—2% and PCLAO—4%) at two different concentrations. CTL—no treatment, control group. DEAD—dead cells, reaction control group. Student *t*-test used for statistical analysis. (*) statistically significant difference (*p* < 0.05) compared with the control group. (#) statistically significant difference (*p* < 0.05) compared with the PCL group.

The 3T3 fibroblasts incubated in the RJS scaffolds of PCL—0, PCLA—2%, PCLA—4%, PCLOA—2%, and PCLOA—4% showed a reduction in cell viability of approximately 2.3%, 72.3%, 76.7%, 63.4%, and 74.5%, respectively, compared with the control group without treatment, considering the value of 100%. All groups with the presence of alginate (with or without pracaxi oil) also showed a reduction of approximately 71.6% (PCLA—2%), 76.1% (PCLA—4%), 62.5% (PCLOA—2%), and 73.9% (PCLOA—4%) compared with the PCL-0 scaffold, considering the value of 100%. As observed in SEM images, the alginate reduced the pores of materials. The porosity influenced the microenvironment and facilitated cell growth and migration within scaffolds [23]. On the other hand, PCL—0 and control (CTL), as well as the PCLA (PCLA—2% and PCLA—4%) and PCLOA (PCOLA—2% and PCLOA—4%) groups, did not show statistically significant differences when compared to each other (Figure 8). Only PCL—0 did not cause cytotoxicity to 3T3 fibroblasts. Polycaprolactone was recognized for its low cytotoxicity. And, the adding of pracaxi oil did not lead to increased cell death. However, recent histological studies have shown that the increase in the dose of pracaxi oil between 0 and 2.400 mg/kg to Rattus norvegicus (lin. Wistar) begins a possible toxic process, such as hyaline and centrilobular degeneration processes and necrosis. And, even though alginate hydrogels are considered, for the most part, biocompatible biopolymeric materials and are widely used in tissue engineering and controlled drug delivery, impurities, composition, roughness, excess calcium ions, and the concentration of the formulation components can result in potential effects of toxicity, as observed in the work of Raddatz et al. (2018) [56]. Thus, the material composition and porosity have a pivotal role in cell viability. Findings have shown significant implications for developing scaffolds in tissue engineering, underscoring the critical importance of selecting starting materials. Choosing mechanically suitable materials for tissue engineering, as well as biocompatibility properties, are imperative to support cell growth and adhesion [57]. This study highlights the necessity for carefully considering additives (vegetable oil, crosslinker, and plasticizer), given their potential to substantially impact cell viability and biocompatibility. Thus, investigating the hydrogel’s formulation components (impurities and dosage) [58] and evaluating the interactions between pracaxi oil and scaffolds could yield insights to mitigate the cytotoxic effects and enhance their biocompatibility as a bioactive scaffold to support cell adhesion and proliferation.

## 4. Conclusions

RJS scaffolds based on PCL and alginate hydrogel (PLCA and PCLOA group) proved an excellent strategy for obtaining multizonal bilayer materials. Contact angle measurements and scanning electron microscopy showed surfaces with wettability and porosity gradients, depending on the alginate and/or pracaxi oil content, respectively. The maximum degradation temperature was also affected by both (ALG and PO). Molecular interactions were observed between all components of the formulation. The PO presented a chemical composition, primarily in oleic, linoleic, and behenic acid (important constituents in cellular signaling and communication for promoting healing and tissue regeneration). It also acted in reducing the crystallinity and closing the pores of the scaffolds of the PCLOA group. Nevertheless, it had no significant influence on cell death, as observed in in vitro assays. The alginate hydrogel’s composition, porosity, or excess of calcium ions may have led to reduced cell viability in the materials, although the polysaccharide favored their hydrophilicity. Thus, this study opens precedents for new investigations with the oil of the Pentaclethra macroloba species in the generation of bioproducts of multizonal architecture for tissue regeneration, even associated with other hydrogels. This promising approach combines biomaterials, hydrogels, and Amazonian bioactives with the potential to stimulate cell migration and proliferation. However, the results emphasize the need for meticulous evaluation of all components within scaffold formulations and suggest the association of PO only to the PCL—0 group, without alginate, to evaluate scaffold biocompatibility and safety for applications in tissue engineering.

## Figures and Tables

**Table 1:** Physicochemical properties of pracaxi oil.

Physicochemical Data	Units	Values *
Density	25 °C g/mL	0.9173
Acidity level	mg NaOH/g	<10.0
Iodine index	gI_2_/100 g	50–77
Peroxide content	10 meq O_2_/Kg	<10.0
Melting point	°C	18.5
Unsaponifiable matter	%	<1.5
Saponification index	mg KOH/g1	175–188

* Data obtained by the Amazon Oil supplier. * MM—molecular mass.

**Table 2:** Identification and composition of samples.

Sample Name	Composition *
PCL—0	PCL 20% (*w*/*v*)
PCLA—2%	PCL 20% (*w*/*v*)/alginate (2% *w*/*v*)
PCLA—4%	PCL 20% (*w*/*v*)/alginate (4% *w*/*v*)
PCLA—6%	PCL 20% (*w*/*v*)/alginate (6% *w*/*v*)
PCLOA—2%	PCL 20% (*w*/*v*)/alginate (2% *w*/*v*), immersed in pracaxi oil
PCLOA—4%	PCL 20% (*w*/*v*)/alginate (4% *w*/*v*), immersed in pracaxi oil
PCLOA—6%	PCL 20% (*w*/*v*)/alginate (6% *w*/*v*), immersed in pracaxi oil

* Calcium chloride concentration at 4% m/v in distilled water.

**Table 3:** Pracaxi oil fatty acid composition.

Nomenclature	Composition (%)	Chain
Lauric acid	0.77	C12:0
Myristic acid	0.72	C14:0
Palmitic acid	2.42	C16:0
Margaric acid	1.44	C17:0
Stearic acid	3.15	C18:0
Oleic acid	53.68	C18:2 (ω-9)
Linoleic acid	12.61	C18:2 (ω-6)
Nonadecanoic Acid	0.14	C19:0
Arachidic Acid	1.34	C20:0
Behenic acid	13.74	C22:0
Tricosanoic Acid	0.12	C23:0
Lignoceric acid	9.82	C24:0

**Table 4:** TGA and DrTGA curve parameters of the scaffolds and pracaxi oil: degradation onset temperature (Tonset), maximum degradation temperature (Tdmax), weight loss (%), and percentage of residues.

Samples	Tonset (°C)			Tdmax (°C)			Total Weight Loss (%)	Residue (%)
	1° Stage	2° Stage	3° Stage	1° Stage	2° Stage	3° Stage		
PCL—0	210.29			406.9			95.16	4.84
PCLA—2%	238.59	424.60		385.77	472.28		96.41	3.59
PCLA—4%	55.15	248.49	385.05	85.9	348.13	451.59	91.74	8.26
PO	190.62	317.08		258.07	420.31		98.31	1.69
PCLOA—2%	197.57	276.66	426.38	225.65	393.46	439.03	97.40	2.60
PCLOA—4%	177.12	284.84	436.59	242.78	379.06	460.56	98.21	1.79

## Data Availability

The data presented in this study are available on request from the corresponding author.
